# The Application of New Molecular Methods in the Investigation of a Waterborne Outbreak of Norovirus in Denmark, 2012

**DOI:** 10.1371/journal.pone.0105053

**Published:** 2014-09-15

**Authors:** Lieke B. van Alphen, Frédérique Dorléans, Anna Charlotte Schultz, Jannik Fonager, Steen Ethelberg, Camilla Dalgaard, Marianne Adelhardt, Jørgen H. Engberg, Thea Kølsen Fischer, Sofie Gillesberg Lassen

**Affiliations:** 1 Department of Microbiological Diagnostics and Virology, Statens Serum Institut, Copenhagen, Denmark; 2 European Programme of Public Health Microbiology (EUPHEM), European Centre for Disease Prevention and Control (ECDC), Stockholm, Sweden; 3 Department of Infectious Disease Epidemiology, Statens Serum Institut, Copenhagen, Denmark; 4 European Programme for Intervention Epidemiology Training (EPIET), European Centre for Disease Prevention and Control (ECDC), Stockholm, Sweden; 5 National Food Institute, Division of Food Microbiology, Danish Technical University (DTU), Lyngby, Denmark; 6 Danish Health and Medicines Authority, Public Health Medical Officers East, Denmark; 7 Regional Clinical Microbiology Laboratory, Region Zealand, Denmark; The University of Melbourne, Australia

## Abstract

In December 2012, an outbreak of acute gastrointestinal illness occurred in a geographical distinct area in Denmark covering 368 households. A combined microbiological, epidemiological and environmental investigation was initiated to understand the outbreak magnitude, pathogen(s) and vehicle in order to control the outbreak. Norovirus GII.4 New Orleans 2009 variant was detected in 15 of 17 individual stool samples from 14 households. Norovirus genomic material from water samples was detected and quantified and sequencing of longer parts of the viral capsid region (>1000 nt) were applied to patient and water samples. All five purposely selected water samples tested positive for norovirus GII in levels up to 1.8×10^4^ genomic units per 200 ml. Identical norovirus sequences were found in all 5 sequenced stool samples and 1 sequenced water sample, a second sequenced water sample showed 1 nt (<0.1%) difference. In a cohort study, including 256 participants, cases were defined as residents of the area experiencing diarrhoea or vomiting onset on 12–14 December 2012. We found an attack rate of 51%. Being a case was associated with drinking tap-water on 12–13 December (relative risk = 6.0, 95%CI: 1.6–22) and a dose-response relation for the mean glasses of tap-water consumed was observed. Environmental investigations suggested contamination from a sewage pipe to the drinking water due to fall in pressure during water supply system renovations. The combined microbiological, epidemiological and environmental investigations strongly indicates the outbreak was caused by norovirus contamination of the water supply system.

## Introduction

Noroviruses (NoV) are considered to be the major cause of gastroenteritis outbreaks and an important cause of sporadic gastroenteritis in both children and adults [1]. In the last decade, NoV genotype II.4 (GII.4) has emerged as the predominant genotype worldwide responsible for the majority of outbreaks and sporadic cases of NoV gastroenteritis [2]–[5]. NoV transmission occurs fecal-orally as viral particles are shed in human stool and vomit [6], via contaminated food or water or via person-to person contact [7]. In a suspected outbreak situation, molecular typing of NoV can provide information on a suspected common source of infection. The molecular typing is performed through genotype assignment and comparisons of polymerase and capsid gene sequences of isolates from infected persons [8], [9]. More recently, comparisons of the hypervariable P2 region of NoV has shown to be an efficient tool for molecular epidemiological investigations of NoV transmission [10], [11].

NoV outbreaks are the most common foodborne outbreaks in Denmark, especially during the winter season where outbreaks have been linked to consumption of various food-items [12]. Waterborne outbreaks are rare in Denmark, with only 5 waterborne outbreaks reported since 1991 [13]–[16] only one of which included NoV. This could be due to the fact that tap-water in Denmark mainly comes from groundwater sources [17], which are rarely contaminated. Although epidemiological evidence can identify water as the outbreak vehicle, it can be difficult to prove contamination by detection of virus in the water [18], [19]. This can be partly attributed to the low viral content of water, which demands efficient purification of viral nucleic acids. Successful determination of NoV commonly requires analysis of large quantities of water. However, a recent waterborne outbreak investigation in China reported finding NoV nucleic acids in well-water samples, using approximately 1 liter water samples [20].

## Outbreak Notification

On 13 December 2012, the water supply company and local health authorities in Kalundborg, a provincial town on Zealand, Denmark, received notifications of gastrointestinal illness among 1) residents in an apartment building and 2) children in a daycare, respectively. Further, on 14 and 15 December, the local medical on-call service and acute medical number received an increased number of calls of gastrointestinal illnesses. This was reported to the local health authorities. On 15 December, laboratory results of water samples taken from the affected apartment building by the water company showed increased indicator bacteria (*Escherichia coli* and coliforms). This was reported to the municipality on the same day. The different lines of information were linked and suggested a possible waterborne outbreak. An outbreak investigation was initiated by the municipality and the first control measures were put in place on 15 December. Statens Serum Institut (SSI) was invited to support the investigation on 17 December. The outbreak team consisted of representatives from Kalundborg municipality, the water supply company, the local health authority, the local food and veterinary authorities, local police and fire departments and SSI.

We conducted microbiological, epidemiological and environmental investigations in the affected area in order to confirm the outbreak, understand its scale, find the source of the contamination, to identify the causative agent and inform public health actions.

## Materials and Methods

### Ethics statement

This study was conducted as an outbreak investigation for which, according to Danish regulations, ethical clearance by the National Committee on Health Research Ethics is not needed; neither for the questionnaire survey nor the collection of fecal material for analysis. However, for the latter, detailed information about the study was given as part of the face-to-face interview and informed consent obtained verbally. Personal data information was saved on a secure server and was only accessible to investigators. The investigation was approved by the Danish Data Protection Agency (record number 2008-54-0474).

### Microbiological investigation of patient and environmental samples

On 17 December 2012, we provided 23 persons with gastrointestinal symptoms from 15 conveniently selected households in the affected area with stool collection packages consisting of a stool container with a spoon, a questionnaire regarding symptoms (onset and duration), a laboratory request form and a pre-stamped return envelope. In addition, stool samples from two other residents living in the affected area, (sampling date 17 December 2014) were located among all samples submitted to the regional clinical microbiological laboratory in December 2013. Samples returned to SSI were tested for a range of virus, bacteria and parasites. Briefly, culturing was performed for pathogenic bacteria, including diarrhoeagenic *E. coli*, *Campylobacter*, *Salmonella* and *Yersinia* [21]. The presence of gastrointestinal viruses was assessed according to previous publications using a multiplex real time PCR for NoV (GI & GII) [22], Rotavirus [23], Sapovirus [24], Human Astrovirus and Human Adenovirus (40+41) [25]. Parasites were diagnosed using RT-PCR and microscopy [26].

Typing of NoV in stool samples was performed by sequencing nested RT-PCR products obtained with primers [27]–[29] able to amplify 337 nt (position 4280–4617 relative to the GU445325.2 reference sequence) of the polymerase and/or with primers [22], [30], [31] able to amplify 341 nt (position 5048–5389 relative to the GU445325.2 reference sequence) of the capsid genesof a broad range of NoV genotypes. For the water samples, only the primers targeting the capsid region was applied. When Norovirus GII.4 was identified in both stool and water samples, further typing was performed using primers that specifically amplify ∼85% (position 5128–6699 relative to the GU445325.2 reference sequence) of the NoV capsid gene as described previously [32]. Phylogenetic analysis of nucleotide sequences was performed in Mega 5 [33] using the Jukes Cantor Model of Substitution and 1000 bootstrap replicates. All sequences were submitted to GenBank.

From 14 December 2012 to 23 January 2013, water samples were collected daily at up to 19 different addresses distributed within the affected area and at five addresses outside of the affected area. In total 421 samples were tested at the local environmental laboratory for indicators of fecal contamination: the number of *E. coli* and coliform colony forming units per 100 milliliter (Colilert SM9223–2005).

Five of the *E. coli* positive water samples were selected for detection, quantification and characterization of pathogens. Sample selection was based on time point as close to the contamination as possible and coming from separate addresses. To analyze for enteric viruses, viral particles were precipitated from 200 ml of water by the addition of PEG (80 g/l) and NaCl (17.5 g/l) before centrifugation at 10.000×g for 90 min. The resulting pellet was resuspended in nucliSens lysis buffer (BioMerieux, Denmark) to digest viral capsids and released nucleic acids were extracted using nucliSens reagens (BioMerieux) according to the manufacturers' protocol. RNA was eluted in 100 µl RNAse-free water. To estimate a potential loss of virus and variability between samples inherent to the procedure for virus recovery, the extraction efficiency of viral RNA was evaluated for each sample. This was done by testing the percentage of recovered mengovirus (strain ATCC VR-1957) [34] added in the amount of 10^4^ pfu as an internal process control virus to each water sample and to a non-matrix sample before the initial step of viral precipitation. Extracted nucleic acids (5 µl undiluted and 10-fold diluted) were separately assayed in duplicate by real-time RT-qPCR for NoV GI and GII [35], sapovirus [36], [37], astrovirus [38], rotavirus [23] and mengovirus [34] using the RNA UltraSense One-step qRT-PCR system (Invitrogen). We used the reaction conditions described in the ISO TS 15216 [39] for NoV GI and GII and mengovirus, and in the manufacturer's protocol, except for using an annealing temperature of 55°C instead of 60°C for the remaining viruses. NoV GI.3b and GII.1 transcripts (5–50 genomic units) or RNA extracts of cell culture grown mengovirus or stool samples positive for sapovirus, astrovirus or rotavirus (5–50 RT-qPCR units of the respective viral genomes), were included in the appropriate assays to act as positive amplification controls, and water was used as negative amplification control. RT-qPCR fluorescence signals resulting in sigmoid curves crossing the threshold line (set to 0.1) above the background signals of non-virus control samples were considered true positives. Detected NoV GI and GII were quantified by interpolation of Ct values to standard curves derived from 5 to 5000 transcripts of GI.3b and GII.1 [40] and the figures were subsequently adjusted for volume changes (uncorrected figures). If the Ct value was outside the linear range of the standard curve, the amount of detected genome units was defined as below theoretical limit of quantification (<tLOQ). The theoretical limit of detection and quantification (100% recovery and amplification efficiency) of the method was determined at 20 or 100 genomic units, respectively, of NoV per 200 ml. The calculated extraction efficiency of viral nucleic acid obtained for each sample was applied to adjust the uncorrected genomic units/200 ml water obtained above (corrected figures).

### Epidemiological investigation

We conducted a retrospective cohort study including residents of the affected water distribution area. Structured questionnaires (a structured questionnaire consisted of a set of one household and seven identical individual questionnaires - one individual questionnaire was filled by each household member) and pre-stamped return envelopes were placed in household mailboxes on 19 December 2012.

We defined a case as a resident of the affected area with onset of diarrhea or vomiting on 12–14 December 2012, as water contamination occurred between 11 and 12 December. Residents not reporting any information on illness status, symptoms or date of onset and residents who had traveled on 11 to 15 December 2012 or who had onset of gastrointestinal symptoms before 12 December were excluded from the analysis. Residents reporting symptom onset after 14 December were excluded from relative risk calculations due to the possibility of being secondary cases.

Cases were described by demographic characteristics, duration and date of disease onset, incubation time, doctor consultation, hospitalization and tap-water consumption.

Water exposure was defined as tap-water consumption on 12–13 December. Daily average tap-water consumption was calculated where information on daily tap-water consumption for at least one of the two days was complete. When information was missing for one day, the daily intake was set to 0.

Attack rates (AR), relative risks (RR) and 95% confidence interval (95% CI) for tap-water consumption were calculated. A Poisson regression with over-dispersion for linear trend for dose-response relation was calculated. Data entry was done using Epidata version 2.0 (EpiData Association, Denmark). Data analysis was performed using Stata 12 (Stata Corp., USA) and Excel 2010 (Microsoft Corporation, USA).

### Environmental investigations

The tap-water supply system was assessed using camera investigations and water flow simulations, to identify water pipelines breakages and possible points of water contamination.

## Results

### Microbiological investigation of human stool samples

Of the 23 distributed stool-sample packages, 17 were returned by members of 14 households. NoV GII was detected in 16 out of 17 (94%) stool samples. VTEC O146 was identified in two NoV positive samples; both from one household. Samples from two additional patients living in the affected area were identified as NoV-positive at the regional clinical microbiological laboratory.

Initial typing of the patient NoV isolates identified the NoV GII.4 New Orleans 2009 variant in all 15 typed patient samples; 6 based on both polymerase and capsid sequences and 9 based on polymerase sequences only (data not shown).

### Microbiological investigation of water samples

The first water sample taken within the affected area on 14 December 2013 proved positive for *E. coli* and coliforms with counts of 130 CFU/100 ml *E. coli* and 50 CFU/100 ml coliforms (Table 1), while water samples outside the affected area remained negative. At least one daily water sample remained positive for indicator bacteria until 21 January 2013.

**Table 1:** Results of positive microbiological findings in water samples.

Water sample	Sampling Date	*E. coli* CFU/100 ml	Coliforms CFU/100 ml		NoV GII genomic units^a^/200 ml		
					Without correction	With correction	
W1	14-12-2012	1	1	1.0×10^3^			5.3×10^3^
W2	14-12-2012	130	50	1.6×10^2^			8.3×10^2^
W3	16-12-2012	10	4	2.2×10^3^			1.8×10^4^
W4	16-12-2012	>200	130	<tLOQ^b^ (7.0×10^1^)			
W5	17-12-2012	3	1	3.7×10^2^			8.4×10^2^

a Estimations are figured with or without applying corrective factors derived by extraction efficiencies.

b Below theoretical limit of quantification estimated at 100 genomic units/200 ml.

Five water samples, W1–W5, were selected for viral analyses. Selection was based on date of collection as close to the initial report of gastrointestinal illness and testing positive for *E. coli* and coliforms. The selected water samples were collected on 14, 16 and 17 December (Table 1). NoV GII was detected in the undiluted RNA extracts from all five water samples. Additionally, for sample W1–W3 and W5 NoV GII could also be detected in the respective 10-fold diluted RNA extracts. Estimated levels of NoV GII fell in the range from <tLOQ (estimated to 100 genome units) to 2.2×10^3^ genomic units per 200 ml of water (Table 1, uncorrected figures). Neither NoV GI, sapovirus, astrovirus nor rotavirus could be detected in any of the water samples. We did not test the extracts directly for assay inhibition during detection, as the limited amount of RNA that could be extracted from the water samples was prioritized for the testing of the mentioned viruses and to sequence the detected NoV GII. By comparing the levels of detected NoV GII as well as the recovered mengovirus in undiluted and 10×diluted extracts, we found up to 1 log assay inhibition in the neat extracts. To more precisely estimate the levels of NoV GII, we therefore used Ct values obtained from 10-fold diluted extracts. The recovery of mengovirus as process control in five water extracts showed an average extraction efficiency of 26% (range 12 to 44%). Applying these factors to adjust the uncorrected NoV GII levels (based on the RT-qPCR assay including volume changes), resulted in corrected amounts of genomic units of NoV GII ranging from <tLOQ to 1.8×10^4^ per 200 ml (Table 1, corrected figures). Partial and/or full capsid sequencing on the water samples was successful for the two water samples (W1 and W3) containing the highest number of NoV genomic units. NoV GII.4 variant New Orleans 2009 was identified in both samples. We were unable to detect and type *E. coli* in the water samples when tested at SSI.

### Comparison of NoV capsid sequences from patients and water samples

**Figure 1:**
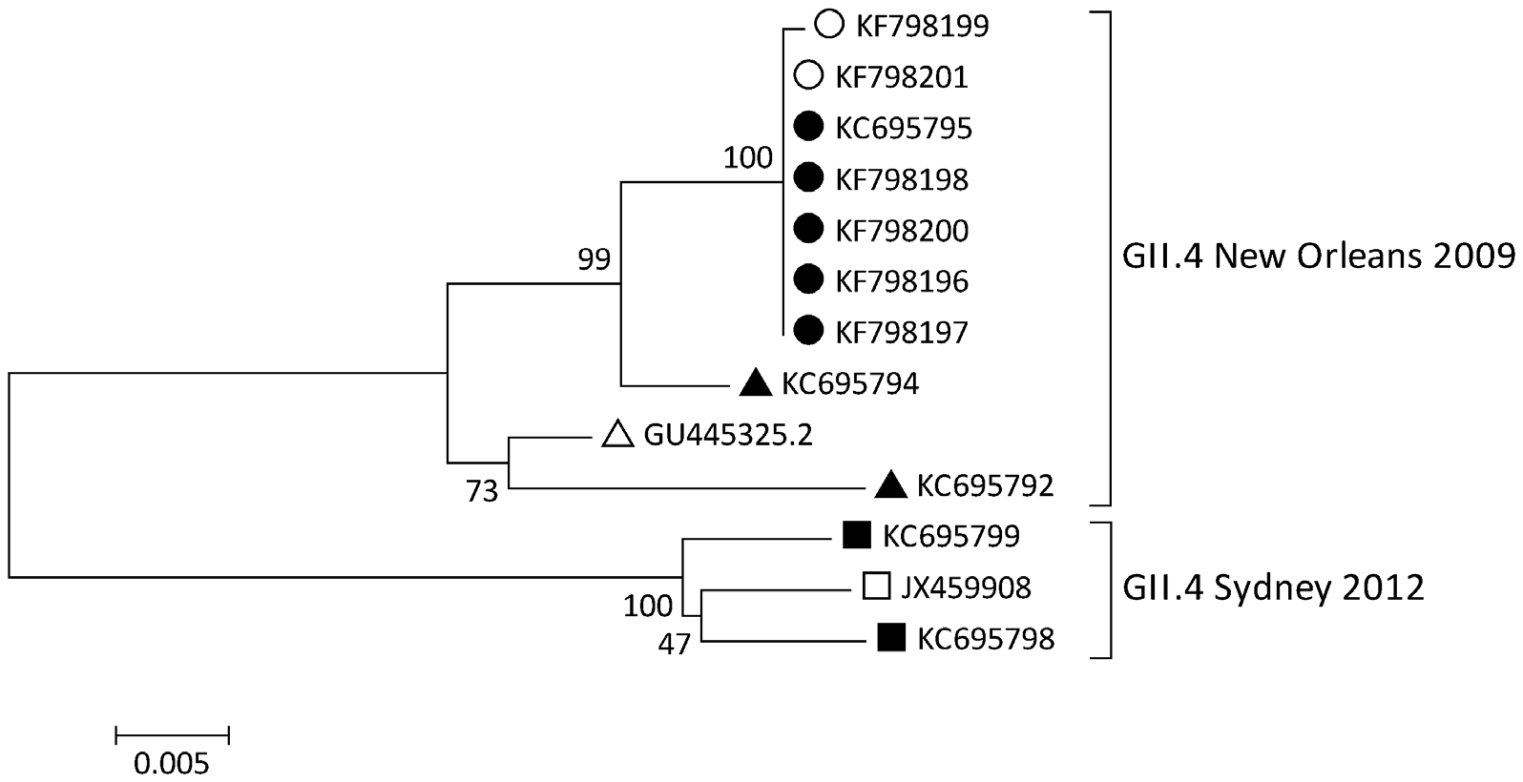
Phylogenetic tree of NoV capsid gene sequences. Phylogenetic tree based on 11 nucleotide sequences of 1140 nt of the NoV capsid gene. All sequences are indicated by their GenBank Accession number. GenBank accession numbers used in this manuscript are from norovirus sequences from patient samples (KF798196, KF798197, KF798198 and KF798200) and water samples (KF798199 (W3) and KF798201 (W1)). The remaining norovirus sequences from patient samples used in the phylogenetic tree have been published previously [32]. Legend: Circles and triangles: New Orleans 2009 variants and Squares: Sydney 2012 variants, Circles: belonging to the outbreak, Triangles: not belonging to the outbreak described here. Open triangle and square are the reference sequences for the New Orleans 2009 and Sydney 2012 variants respectively. Open circles:sequences obtained from water samples (KF798201,W1) and KF798199,W3) and closed circles are the sequences obtained from patient stool samples.

Phylogenetic analysis of five capsid sequences from patient isolates originating from different households in this outbreak was compared to four Danish capsid reference sequences belonging to either the New Orleans 2009 or the Sydney 2012 variant [32]. This analysis showed clustering of sequences from all five human samples and also with the two water samples (Figure 1). All patient samples and one water sample (W1) were 100% identical, whereas W3 water sample differed by 1 nucleotide (out of the 1140 nt sequences shared between all patient and water samples, ∼0.1%).

### Epidemiological investigation

A structured questionnaire was sent to 368 households of which 154 responded (response rate: 41.8%). In total 276 individual questionnaires were received from the 154 households. We excluded 20 individuals from eighteen households based on the exclusion criteria.

**Figure 2:**
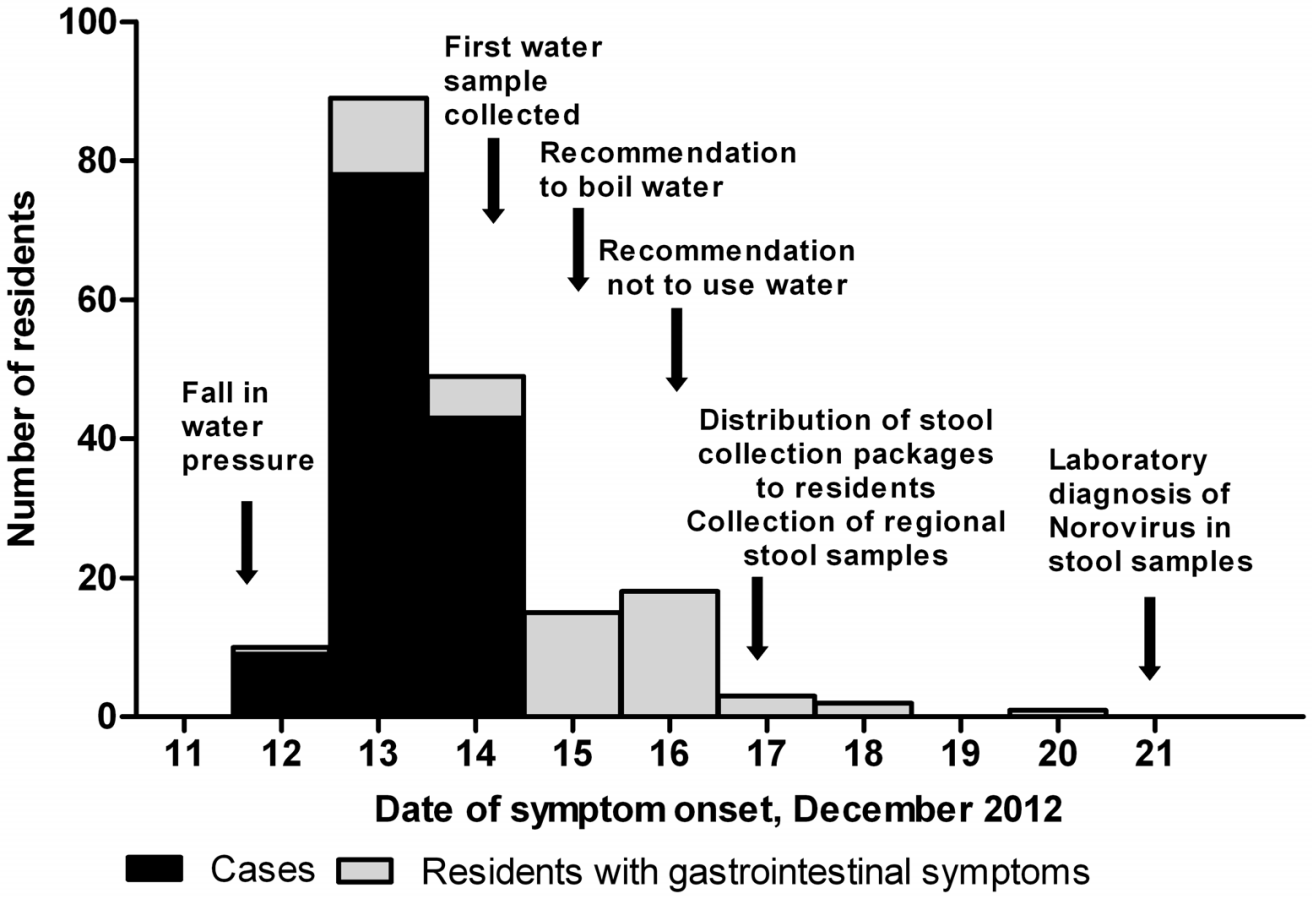
Date of onset and case status of individuals with gastrointestinal symptoms. Number of individuals in the cohort with gastrointestinal symptoms by date of symptom onset and case status (N = 187), 12–21 December 2012, Kalundborg, Denmark. Individuals not fulfilling the inclusion criteria were excluded from the cohort. Individuals with gastrointestinal symptoms, but not fulfilling the case definition are indicated as residents with gastrointestinal symptoms. Arrows indicate important events in the outbreak investigation.

Symptoms reported included nausea (61%), diarrhoea (54%), abdominal pain (54%), vomiting (52%), headache (46%), fever (31%) and bloody diarrhea (2%). A total of 130 respondents fulfilled the case-definition (Attack Rate (AR) = 51%). Of the 146 households, 88 (60%) had at least one case. For cases, the median age was 58 years (range: 1–86 years) and 55% were female. The attack rate was 59% for both females and males. The median duration of illness for cases was three days and 78 (60%) cases had symptom onset on 13 December (Figure 2). No case was hospitalized, however, 24 (18%) reported consulting a doctor. The median incubation time was 45 hours (range: 3–69) after the drop in water pressure in the night of 11–12 December.

The ARs for individuals drinking tap-water and individuals not drinking tap-water on 12–13 December were 66% and 11%, respectively, giving a relative risk (RR) of 6.0, 95%CI = 1.6 – 22 (Table 2).

**Table 2:** Attack rates (AR),relative risk (RR) and 95% confidence interval (CI) of diarrhoea and/or vomiting compared to the reference (Ref) on 12–14 December by tap-water consumption and mean daily tap-water consumption on 12–13 December, Kalundborg, Denmark.

		Cases	Total	AR (%)	RR	95% CI
Tap water consumption	Yes	127	192	66	6.0	1.6 – 22
	No	2	18	11	Ref	-
Mean daily number of glasses	0–1	25	67	37	Ref	-
	1.5–2	18	30	60	1.6	1.1–2.5
	2.5–3	22	34	65	1.7	1.2–2.6
	≥3.5	65	90	72	1.9	1.4–2.7

The attack rate increased significantly with increasing consumption of tap water, from 37% for 0–1 mean glasses consumed to 72% for 3.5 or more mean glasses consumed on 12–13 December (Table 2) showing a dose-response relation for mean glasses of tap-water consumed (IRR = 1.07, 95%CI: 1.03 – 1.10 and p-value<0.001).

### Environmental investigation

**Figure 3:**
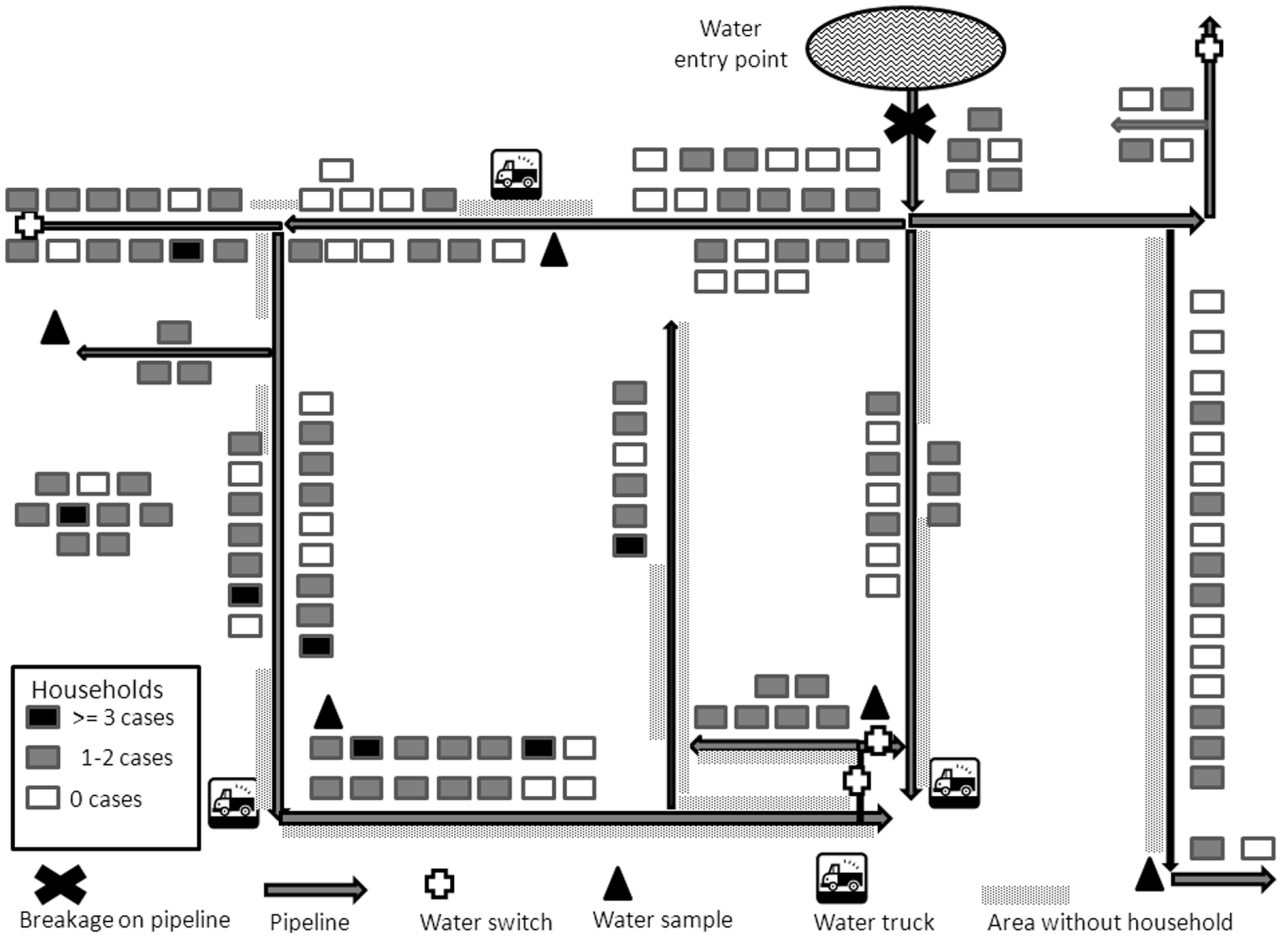
Number and distribution of cases by households in the affected water supply area. Number and distribution of cases by households in the affected water supply area, 12–14 December 2012, Kalundborg, Denmark.

On 19 December, camera investigation identified a breakage of a tap-water pipeline at a location where a leaking sewage pipe was crossing in close proximity above it. The leakage could not be identified from above the ground. A decrease in pressure in the water pipes between 11–12 December could allow sewage water to enter the tap-water supply system (Figure 3).

### Control measures

Valves in the tap-water supply system restricted the contamination to a limited distribution area. A recommendation to boil water before consumption was issued on 15 December. This was communicated via the websites of the municipality and water supply company, in newspapers, radio and television broadcasts, letters to all residents as well as text messages to all mobile phone owners in the affected area. Restaurants and child care institutions located in the contaminated area were closed following the alert. On December 16, due to the reports of a higher than usual number of calls on gastrointestinal symptoms to the medical acute numbers a recommendation not to use any tap-water (water-ban) was issued and the waterworks started to flush the water pipes in order to remove the contamination. Distribution of clean water was organized by the local fire department with three water trucks stationed in the contaminated area and delivering clean water to the residents, from 6am to 11pm every day (Figure 3). The water-ban was changed to a boiling recommendation on 23 December when *E. coli* was found in only four of 13 water samples and the *E. coli* count was 1 in three samples and 4 in one sample. Water samples were analysed daily until the levels of *E. coli* and coliforms were below detection for 2 days. The recommendation to boil water was lifted on 23 January 2013.

## Discussion

We present the microbiological, epidemiological and environmental investigations of an outbreak of NoV-associated gastroenteritis in a geographic area covering 368 households. All investigations point to a one-time contamination of the drinking water supply; indistinguishable types of identical NoVs were detected in drinking water as well as in stool samples of residents from the affected area and cohort study indicated a point source outbreak and showed statistical significant increased risk of diarrhea and/or vomiting for participants who consumed tap-water on 12–13 December and an incubation time corresponding with norovirus infection.

This is, to our knowledge, the first report on a long (>1000 nt) NoV capsid sequence being obtained from an environmental sample and used to assess the sequence identity between NoV in patients and in the immediate environment. Detection of NoV in water samples is difficult, partly due to the large quantities of water (around 5 L) usually needed with previously described methods [41]. The simple method described here demonstrated that 200 ml was enough for efficient virus detection.

NoV sequencing can effectively be used in outbreak investigations. Typing of the NoV P2 region has proven to be highly informative for molecular epidemiological investigations of NoV transmission, as not all genome regions give sufficient resolution to distinguish between certain GII.4 variants, especially in outbreak settings [42]. Analysis of NoV polymerase and/or capsid sequences to determine the genotype and designate a variant showed that all patient samples contained the NoV GII.4 New Orleans 2009 variant. For higher resolution and increased sensitivity, we sequenced 1140 nt of the capsid region (including the hypervariable P2 region) of five patient samples from separate households and two water samples using a recently described method [32]. Interestingly, 100% identical NoV sequences were found in all five patient samples, strongly indicating a common source of infection. A comparison to the NoV sequences from the tap water showed that the NoV sequence in one water sample was identical to that observed in the patient samples and another water sample showed 1 nt (<0.01%) difference, providing evidence that the source of the outbreak most likely was contamination of tap-water at one point in time. Additionally, NoV typing primers, capable of detecting several NoV genotypes only detected NoV GII.4, while outbreaks associated with contamination with sewage water can involve multiple strains of different genotypes or even multiple pathogens [28]. A recent review of a large number of NoV outbreaks showed that waterborne outbreaks are significantly associated with GI strains while GII strains tend to be associated with healthcare-related and winter outbreaks [43], [44]. However, waterborne outbreaks with GII.4 NoV have been reported before [20].

The NoV GII.4 New Orleans 2009 variant was the single most dominant NoV variant in Denmark during 2011 and until October 2012, but was being replaced towards the end of 2012 by the recently emerging 2012 Sydney variant [32]. The molecular analysis used in this study showed that typing and sequence comparison of larger parts of the NoV capsid gene can provide a strong molecular-epidemiological-link between infected persons and suspected environmental sources of infection, provided sufficiently clean extracts.

The water contamination with NoV was only detected after additional testing at a specialized laboratory. The method chosen to extract viruses from water was based on the limited volume (200 ml) of a single portion of polluted water collected at different sampling points in the pipe system. Considerations to minimize virus loss and matrix effects made us use direct virus precipitation based on PEG, thus minimizing limitations in recovery due to filtration [45] and in amplification due to addition of proteins [46]. By following the viral recovery using an internal process control and optimizing the amplification efficiency by testing and evaluating results from undiluted and diluted extracts, we found viral recoveries in the range of what previous studies have shown, 1.5–45% [20], [47], [48] and could thereby limit (by up to approximately one log) inhibition interfering with detection and quantification. Recently, it has been suggested that enteric viruses could act as molecular markers for water quality, as the monitoring of single indicator organisms such as bacteriophages and *E. coli* do not correlate with pathogens, demonstrating that public health might not be adequately protected using traditional monitoring schemes [49]. In this outbreak, the first water sample was taken after illness occurred and thus several days after contamination of the water. The late sampling did not interfere with the detection and further analysis of NoV in the water. However, given that the water samples could not be transported to the diagnostic facilities at the SSI before 20 December and that the SSI is not equipped to routinely analyse water we were not able to detect and type *E. coli* in the water samples. The *E. coli* O147 observed in two patients from the same household may therefore theoretically also have been present in the water and might have contributed to the gastrointestinal symptoms in other patients. However, the fact that the two *E. coli* positive patients were from the same household and that *E. coli* was not found in stools of other persons, suggests that the two constituted a separate household outbreak where water was not the source and therefore we consider this an unlikely scenario.

Diarrhea, vomiting, abdominal pain and nausea were the most frequently reported symptoms. The age distribution of cases and attack rates reflects a wide exposure to tap-water. The statistical significant increased risk (RR = 6.0) and dose-response relation between having diarrhoea and/or vomiting on 12–14 when having drunk tap-water on 12–13 December combined with a median incubation time of 45 hours supports a waterborne point-source outbreak with NoV as the causal agent. A dose-response effect for drinking water contaminated with NoV and developing gastrointestinal symptoms has previously been reported [50], [51].

The environmental investigation revealed a leak from a sewage pipe placed above a broken tap-water pipe. The fall in water pressure during water supply renovations on the night of 11 December briefly enabled sewage water contamination of the tap-water supply. Water contamination with sewage water has previously been reported in waterborne outbreaks [41], [52], [53] and a cohort study conducted in Norway showed that breaks and maintenance in water supply system caused an increased risk of gastrointestinal illness among water recipients [54]. The time of contamination is supported by the temporal distribution of cases and average incubation time.

Overall, this investigation showed the benefits of timely cross-sectional collaboration in an outbreak investigation. Initial public health measures were implemented on 15 December and were continuously informed by the microbiological, epidemiological and environmental investigations. On 24 January, a public meeting was organized to inform the citizens about the process and conclusion of the outbreak investigation and to address any questions and concerns about the water safety and outbreak management. It could be concluded that the outbreak affected a large number of people and that the vehicle and pathogen was waterborne NoV, and caused by a temporary one-point sewage contamination of the drinking water due to fall in the pipe water pressure. The application of new and highly sensitive virology methods helped generate solid evidence of the vehicle and pathogen involved.

## References

[1] Glass RI, Parashar UD, Estes MK (2009) Norovirus gastroenteritis. N Engl J Med 361: 1776–85. 19864676 10.1056/NEJMra0804575 PMC3880795

[2] Medici MC, Martinelli M, Abelli LA, Ruggeri FM, Bartolo I Di, et al (2006) Molecular Epidemiology of Norovirus Infections in Sporadic Cases of Viral Gastroenteritis Among Children in Northern Italy. J Med Virol 78: 1486–1492. 16998898 10.1002/jmv.20723

[3] Siebenga JJ, Vennema H, Zheng D-P, Vinjé J, Lee BE, Pang X-L, et al (2009) Norovirus illness is a global problem: emergence and spread of norovirus GII.4 variants, 2001–2007. J Infect Dis 200: 802–12. 19627248 10.1086/605127

[4] Ferreira MSR, Victoria M, Vieira CB, Xavier MPTP, Fioretti JM (2010) Surveillance of Norovirus Infections in the State of Rio De Janeiro, Brazil 2005 – 2008. J Med Virol 82: 1442–1448. 20572088 10.1002/jmv.21831

[5] Dai Y, Hu G, Zhang X, Song C, Xiang W, et al (2011) Molecular epidemiology of norovirus gastroenteritis in children in Jiangmen, China, 2005–2007. Arch Virol 156: 1641–6. 21562879 10.1007/s00705-011-1010-3

[6] Atmar RL, Opekun AR, Gilger M a, Estes MK, Crawford SE, et al (2008) Norwalk virus shedding after experimental human infection. Emerg Infect Dis 14: 1553–7. 18826818 10.3201/eid1410.080117 PMC2609865

[7] Parashar UD, Monroe SS (2001) “ Norwalk-like viruses ” as a cause of foodborne disease outbreaks. Rev Med Virol 11: 243–252. 11479930 10.1002/rmv.321

[8] Vinje J, Vennema H, Maunula L, Bonsdorff C Von, Hoehne M, et al (2003) International Collaborative Study To Compare Reverse Transcriptase PCR Assays for Detection and Genotyping of Noroviruses. J Clin Microbiol 41: 1423–1433. 12682125 10.1128/JCM.41.4.1423-1433.2003 PMC153862

[9] Kroneman a, Vennema H, Deforche K, v d Avoort H, Peñaranda S, et al (2011) An automated genotyping tool for enteroviruses and noroviruses. J Clin Virol 51: 121–5. 21514213 10.1016/j.jcv.2011.03.006

[10] Sukhrie FH a, Beersma MFC, Wong A, van der Veer B, Vennema H, et al (2011) Using molecular epidemiology to trace transmission of nosocomial norovirus infection. J Clin Microbiol 2011 49: 602–6. 10.1128/JCM.01443-10 PMC3043516 21159934

[11] Xerry J, Gallimore CI, Iturriza-Gómara M, Allen DJ, Gray JJ (2008) Transmission events within outbreaks of gastroenteritis determined through analysis of nucleotide sequences of the P2 domain of genogroup II noroviruses. J Clin Microbiol 46: 947–53. 18216210 10.1128/JCM.02240-07 PMC2268335

[12] Anonymous (2012) Annual Report on Zoonoses in Denmark 2012, National Food Institute, Technical University of Denmark. 2012.

[13] Engberg J, Gerner-smidt P, Scheutz F, Møller Nielsen E, et al (1998) Water-borne Campylobacter jejuni infection in a Danish town–a 6-week continuous source outbreak. Clin Microbiol Infect 4: 648–656. 11864264 10.1111/j.1469-0691.1998.tb00348.x

[14] Vestergaard L, Olsen K, Stensvold R, Böttiger B, Adelhardt M, et al (2007) Outbreak of severe gastroenteritis with multiple aetiologies caused by contaminated drinking water in Denmark, January 2007. Eurosurveillance 12: E070329.1. 10.2807/esw.12.13.03164-en 17439795

[15] Laursen E, Mygind O, Rasmussen B, Rønne T (1994) Gastroenteritis: a waterborne outbreak affecting 1600 people in a small Danish town. J Epidemiol Community Health 48: 453–8. 7964354 10.1136/jech.48.5.453 PMC1060007

[16] Gubbels S-M, Kuhn KG, Larsson JT, Adelhardt M, Engberg J, et al (2012) A waterborne outbreak with a single clone of Campylobacter jejuni in the Danish town of Køge in May 2010. Scand J Infect Dis 44: 586–94. 22385125 10.3109/00365548.2012.655773

[17] Geological Survey of Denmark and Greenland (GEUS): Water Supply in Denmark. Available: http://www.geus.dk/program-areas/water/denmark/vandforsyning_artikel.pdf.

[18] Vantarakis A, Mellou K, Spala G, Kokkinos P, Alamanos Y (2011) A gastroenteritis outbreak caused by noroviruses in Greece. Int J Environ Res Public Health 8: 3468–78. 21909318 10.3390/ijerph8083468 PMC3166754

[19] Maunula L, Miettinen IT, von Bonsdorff C-H (2005) Norovirus outbreaks from drinking water. Emerg Infect Dis 11: 1716–21. 16318723 10.3201/eid1111.050487 PMC3367355

[20] Zhou X, Li H, Sun L, Mo Y, Chen S, et al (2012) Epidemiological and molecular analysis of a waterborne outbreak of norovirus GII.4. Epidemiol Infect 140: 2282–9. 22400795 10.1017/S0950268812000374 PMC3487484

[21] Persson S, de Boer RF, Kooistra-Smid AMD, Olsen KEP (2011) Five commercial DNA extraction systems tested and compared on a stool sample collection. Diagn Microbiol Infect Dis 69: 240–4. 21353945 10.1016/j.diagmicrobio.2010.09.023

[22] Kageyama T, Kojima S, Shinohara M, Uchida K, Fukushi S, et al (2003) Broadly Reactive and Highly Sensitive Assay for Norwalk-Like Viruses Based on Real-Time Quantitative Reverse Transcription-PCR. J Clin Microbiol 41: 1548–1557. 12682144 10.1128/JCM.41.4.1548-1557.2003 PMC153860

[23] Pang XL, Lee B, Boroumand N, Leblanc B, Preiksaitis JK, et al (2004) Increased detection of rotavirus using a real time reverse transcription-polymerase chain reaction (RT-PCR) assay in stool specimens from children with diarrhea. J Med Virol 72: 496–501. 14748075 10.1002/jmv.20009

[24] Hansman GS, Ishida S, Yoshizumi S, Miyoshi M, Ikeda T, et al (2007) Recombinant sapovirus gastroenteritis, Japan. Emerg Infect Dis 13: 786–8. 18044044 10.3201/eid1305.070049 PMC2738451

[25] Logan C, O'Leary JJ, O'Sullivan N (2006) Real-time reverse transcription-PCR for detection of rotavirus and adenovirus as causative agents of acute viral gastroenteritis in children. J Clin Microbiol 44: 3189–95. 16954246 10.1128/JCM.00915-06 PMC1594742

[26] Stensvold CR, Nielsen HV (2012) Comparison of microscopy and PCR for detection of intestinal parasites in Danish patients supports an incentive for molecular screening platforms. J Clin Microbiol 50: 540–1. 22090410 10.1128/JCM.06012-11 PMC3264173

[27] Vennema H, Bruin E De, Koopmans M (2002) Rational optimization of generic primers used for Norwalk-like v irus detection by re v erse transcriptase polymerase chain reaction. J Clin Virol 25: 233–235. 12367660 10.1016/s1386-6532(02)00126-9

[28] Le Guyader F, Neill FH, Estes MK, Monroe SS, Ando T, et al (1996) Detection and analysis of a small round-structured virus strain in oysters implicated in an outbreak of acute gastroenteritis. Appl Environ Microbiol 62: 4268–72. 8900022 10.1128/aem.62.11.4268-4272.1996 PMC168251

[29] Hoebe CJP a, Vennema H, de Roda Husman AM, van Duynhoven YTHP (2004) Norovirus outbreak among primary schoolchildren who had played in a recreational water fountain. J Infect Dis 189: 699–705. 14767824 10.1086/381534

[30] Kojima S, Kageyama T, Fukushi S, Hoshino FB, Shinohara M, et al (2002) Genogroup-specific PCR primers for detection of Norwalk-like viruses. J Virol Methods 100: 107–14. 11742657 10.1016/s0166-0934(01)00404-9

[31] Gallimore CI, Cheesbrough JS, Lamden K, Bingham C, Gray JJ (2005) Multiple norovirus genotypes characterised from an oyster-associated outbreak of gastroenteritis. Int J Food Microbiol 103: 323–30. 15967530 10.1016/j.ijfoodmicro.2005.02.003

[32] Fonager J, Hindbæk LS, Fischer TK (2005) Rapid emergence and antigenic diversification of the norovirus 2012 Sydney variant in Denmark, October to December, 2012. Euro Surveill 18: 10–13. 23470017

[33] Tamura K, Peterson D, Peterson N, Stecher G, Nei M, et al (2011) MEGA5: molecular evolutionary genetics analysis using maximum likelihood, evolutionary distance, and maximum parsimony methods. Mol Biol Evol 28: 2731–9. 21546353 10.1093/molbev/msr121 PMC3203626

[34] Costafreda MI, Bosch A, Pintó RM (2006) Development, evaluation, and standardization of a real-time TaqMan reverse transcription-PCR assay for quantification of hepatitis A virus in clinical and shellfish samples. Appl Environ Microbiol 72: 3846–55. 16751488 10.1128/AEM.02660-05 PMC1489592

[35] Le Guyader FS, Parnaudeau S, Schaeffer J, Bosch A, Loisy F, et al (2009) Detection and quantification of noroviruses in shellfish. Appl Environ Microbiol 75: 618–24. 19047383 10.1128/AEM.01507-08 PMC2632116

[36] Oka T, Katayama K, Hansman GS, Kageyama T, Ogawa S, et al (2006) Detection of Human Sapovirus by Real-Time Reverse Transcription-Polymerase Chain Reaction. J Med Virol 78: 1347–1353. 16927293 10.1002/jmv.20699

[37] Johnsen CK, Midgley S, Böttiger B (2009) Genetic diversity of sapovirus infections in Danish children 2005–2007. J Clin Virol 46: 265–9. 19695950 10.1016/j.jcv.2009.07.008

[38] Le Cann P, Ranarijaona S, Monpoeho S, Le Guyader F, Ferré V (2004) Quantification of human astroviruses in sewage using real-time RT-PCR. Res Microbiol 155: 11–5. 14759703 10.1016/j.resmic.2003.09.013

[39] Anonymous (2013) ISO/TS 15216-1:2013 Microbiology of Food and Animal Feed – Horizontal Method for Determination of Hepatitis A Virus and Norovirus in Food Using Real-Time RT-PCR – Part 1: Method for Quantification, Part 2: Method for Quantitative Determination.

[40] Gentry J, Vinjé J, Lipp EK (2009) A rapid and efficient method for quantitation of genogroups I and II norovirus from oysters and application in other complex environmental samples. J Virol Methods 156: 59–65. 19041894 10.1016/j.jviromet.2008.11.001

[41] Werber D, Lausević D, Mugosa B, Vratnica Z, Ivanović-Nikolić L, et al (2009) Massive outbreak of viral gastroenteritis associated with consumption of municipal drinking water in a European capital city. Epidemiol Infect 137: 1713–20. 19534843 10.1017/S095026880999015X

[42] Vinjé J, Hamidjaja R, Sobsey MD (2004) Development and application of a capsid VP1 (region D) based reverse transcription PCR assay for genotyping of genogroup I and II noroviruses. J Virol Methods 116: 109–117. 14738976 10.1016/j.jviromet.2003.11.001

[43] Matthews J, Dickey B, Miller R, Felzer J, Dawson B (2012) The epidemiology of published norovirus outbreaks: a systematic review of risk factors associated with attack rate and genogroup. Epidemiol Infect 140: 1161–1172. 22444943 10.1017/S0950268812000234 PMC3350621

[44] Lysén M, Thorhagen M, Brytting M, Hjertqvist M, Andersson Y (2009) Genetic diversity among food-borne and waterborne norovirus strains causing outbreaks in Sweden. J Clin Microbiol 47: 2411–8. 19494060 10.1128/JCM.02168-08 PMC2725682

[45] Di Pasquale S, Paniconi M, Auricchio B, Orefice L, Schultz a C, et al (2010) Comparison of different concentration methods for the detection of hepatitis A virus and calicivirus from bottled natural mineral waters. J Virol Methods 165: 57–63. 20100516 10.1016/j.jviromet.2010.01.003

[46] Borchardt MA, Kieke BA, Spencer SK (2013) Ranking filter methods for concentrating pathogens in lake water. Appl Environ Microbiol 79: 5418–9. 23926086 10.1128/AEM.01430-13 PMC3753953

[47] Victoria M, Guimarães F, Fumian T, Ferreira F, Vieira C, et al (2009) Evaluation of an adsorption-elution method for detection of astrovirus and norovirus in environmental waters. J Virol Methods 156: 73–6. 19056426 10.1016/j.jviromet.2008.11.003

[48] Haramoto E, Katayama H, Oguma K, Ohgaki S (2007) Recovery of naked viral genomes in water by virus concentration methods. J Virol Methods 142: 169–73. 17321606 10.1016/j.jviromet.2007.01.024

[49] Harwood VJ, Levine AD, Scott TM, Chivukula V, Lukasik J, et al (2005) Validity of the Indicator Organism Paradigm for Pathogen Reduction in Reclaimed Water and Public Health Protection Validity of the Indicator Organism Paradigm for Pathogen Reduction in Reclaimed Water and Public Health Protection. Appl Environ Microbiol 71: 3163–31. 15933017 10.1128/AEM.71.6.3163-3170.2005 PMC1151840

[50] Ter Waarbeek HLG, Dukers-Muijrers NHTM, Vennema H, Hoebe CJP (2010) Waterborne gastroenteritis outbreak at a scouting camp caused by two norovirus genogroups: GI and GII. J Clin Virol 47: 268–72. 20056481 10.1016/j.jcv.2009.12.002

[51] Carrique-Mas J, Andersson Y, Petersén B, Hedlund KO, Sjögren N, et al (2003) A norwalk-like virus waterborne community outbreak in a Swedish village during peak holiday season. Epidemiol Infect 131: 737–44. 12948374 10.1017/s0950268803008604 PMC2870015

[52] Kuusi M, Nuorti JP, Maunula L, Minh Tran NN, Ratia M, et al (2002) A prolonged outbreak of Norwalk-like calicivirus (NLV) gastroenteritis in a rehabilitation centre due to environmental contamination. Epidemiol Infect 129: 133–8. 12211580 10.1017/s0950268802007276 PMC2869858

[53] Laine J, Huovinen E, Virtanen MJ, Snellman M, Lumio J, et al (2010) An extensive gastroenteritis outbreak after drinking-water contamination by sewage effluent, Finland. Epidemiol Infect 1–9. 10.1017/S0950268810002141 20843387

[54] Nygård K, Wahl E, Krogh T, Tveit OA, Bøhleng E, et al (2007) Breaks and maintenance work in the water distribution systems and gastrointestinal illness: a cohort study. Int J Epidemiol 36: 873–80. 17389718 10.1093/ije/dym029

